# Production of Bi-Compartmental Tablets by FDM 3D Printing for the Withdrawal of Diazepam

**DOI:** 10.3390/pharmaceutics15020538

**Published:** 2023-02-06

**Authors:** Joana Macedo, Rita Marques, Chris Vervaet, João F. Pinto

**Affiliations:** 1 iMed.ULisboa, Faculdade de Farmácia, Universidade de Lisboa, 1649-003 Lisboa, Portugal; 2 Laboratory of Pharmaceutical Technology, Ghent University, 9000 Ghent, Belgium

**Keywords:** 3D printing, benzodiazepines, bi-compartmental tablet, diazepam, withdrawal, fused deposition modelling, PVA

## Abstract

Diazepam (DZP) is a long-acting benzodiazepine to treat anxiety or acute alcohol withdrawal. Although this class of drugs should be taken for a short period of time, many patients take them for longer than recommended, which has been linked to an increased risk of dementia and dependence. The present work aimed at using the dual-nozzle system of fused deposition modeling (FDM) 3D printers to prepare tablets with gradual doses of DZP with constant mass and size. Placebo and DZP-loaded filaments were prepared by hot-melt extrusion and used to print the bi-compartmental tablets. Thermal processing allowed the conversion of crystalline DZP to its amorphous counterpart. Tablets with different DZP contents were effectively printed with a mass, thickness and diameter average of 111.6 mg, 3.1 mm, and 6.4 mm, respectively. Microscopic data showed good adhesion between the different layers in the printed tablets. The desired drug contents were successfully achieved and were within the acceptance criteria (European Pharmacopeia). The combination of a placebo and drug-loaded extrudates proved to be beneficial in the production of tablets by FDM for patients in need of drug withdrawal.

## 1. Introduction

Diazepam (DZP) is a long-acting (i.e., with a half-life > 24 h) benzodiazepine (BZD) prescribed for the treatment of anxiety, acute alcohol withdrawal, skeletal muscle spasm, and convulsive disorders [1,2]. This class of drugs has a complex action that enhances the efficacy of the neurotransmitter gamma-aminobutyric acid (GABA) at the GABA-A receptor, generating a sedative, hypnotic, anxiolytic, anticonvulsant, and muscle relaxant effect on patients [2,3]. Based on the long-term development of tolerance, dependence, abuse and withdrawal syndrome, BZDs should be used for a maximum duration of 2 to 4 weeks for insomnia or anxiety and no more than 2 weeks for mixed anxiety-depressive disorders [4]. However, long-term use of BZDs is prevalent among patients despite the risks [3,4,5], without receiving first-line treatments, such as psychotherapy or serotonergic-directed agents [5].

Furthermore, DZP has been used to help the withdrawal of short-acting BZDs (i.e., with a half-life < 12 h, like triazolam), especially for patients with both alcohol and BZD dependence, as those are normally associated with worse withdrawal symptoms [2,5]. Recent investigations suggest that the use of BDZs may be associated with a higher risk of dementia, particularly in people using long-acting BDZs for long periods of time [2,6]. Therefore, withdrawal of DZP is required and can be achieved by slowly tapering the dose to overcome the dependency without the appearance of abstinence symptoms [5]. For patients taking less than 20 mg daily, a reduction of 1 mg every 1–2 weeks is acceptable. When taking higher doses (20–40 mg), patients usually tolerate reductions of 2 mg every 1–2 weeks down to a daily dose of 4–5 mg. Thereafter further reductions of 0.5 mg every 1–2 weeks are better tolerated [5]. DZP tablets can be found on the market with discrete potencies of 2, 5 and 10 mg. Although these doses are suitable for proper therapy management [5], when tapering the DZP dose, patients are required to split the tablets into halves or quarters. This procedure remains non-satisfactory because the dose remains discrete; patients are likely to lose part of the tablet resulting in a random BZD dose or difficulties for impaired mobility patients.

As three-dimensional (3D) printing can be used to personalize the drug doses required by the patients, this technique is acceptable for the withdrawal of BZDs. 3D printing enables small-scale production adequate for a continuous adjustment of the dose of active pharmaceutical ingredients (APIs) in a single printing cycle by changing the size or geometry of the printed tablets [7]. Fused deposition modeling (FDM) is a hot-melt extrusion-based 3D printing technology allowing a flexible, cost- and time-efficient production of tablets [7,8], particularly useful for dose individualization. Previous studies have highlighted the usefulness of FDM in assisting the withdrawal of BZDs. For instance, Henry and co-workers studied the dose reduction of zolpidem (ZHT). To achieve this reduction, filaments with different drug loads (1 and 10%) were produced and used to print caplets with different sizes. Furthermore, to change the drug release rate, channeled caplets were prepared [8]. A similar strategy was used by Obeid and co-workers to modify the drug content and release profile of diazepam. Filled cylindrical tablets and tablets with different numbers of holes were prepared using a single filament containing 2.5% DZP [9]. Although these approaches allowed us to achieve different drug contents in tablets, they required the production of different filaments with different drug loads, increasing the workload before the printing process, tablets were visually different (size and/or number of holes) and varied mass (by changing the infill degree) negatively impacting the perception, acceptability and compliance to therapy by patients.

Using FDM 3D printer with a dual nozzle system, a different approach can be considered. Previous research has considered the dual nozzle printer to produce bilayer tablets with different drugs in each layer [10,11,12,13]. However, when using a placebo filament in combination with a drug-loaded filament, it is possible to obtain different drug loads while keeping the total size and mass of tablets constant. Okwuosa and co-workers used a dual-nozzle system to produce gastric-resistant tablets by using an enteric filament in one nozzle to print the shell of the tablet and a drug-loaded filament on the other nozzle to print the core of the tablet [14]. To control the drug release, Tagami et al., in a different study, prepared composite tablets consisting of a drug (calcein) and a filler (either PVA or PLA) compartments [15]. On the other hand, Buyukgoz and co-workers explored different tablet design options for the simultaneous tailoring of the drug release and dose [16] by a judicious balance between placebo and drug-loaded filaments. The authors studied the impact on the release of a model drug by preparing dual tablets in which the drug was present either in the core (placebo as a coat) or in the coat (placebo in the core). Different drug contents were achieved by changing the size of the core, and the different designs led to significant differences in drug release.

Another group of patients that needs special attention and requires specific considerations when designing drug products is pediatric patients. For effective pharmacotherapy, clinical safety and medication adherence are of utmost importance to achieve the desired treatment. In this case, 3D printing extrusion-based technologies, namely FDM [17,18,19,20], semi-solid extrusion [21] and direct powder extrusion [18,22], have been shown to play an important role in the production of personalized medicines for this group of patients.

By using the dual-nozzle system of a 3D printer, the present work aimed to study the use of FDM for the production of tablets with gradual DZP doses (e.g., 10.0, 9.8, 9.7, and 9.55) while maintaining the same appearance (constant size) and mass of tablets, preventing patients of becoming aware of the dose reduction over the treatment time. In this respect, the range of achievable drug contents in tablets was studied while confirming the short-term storage stability of the filaments and tablets made of poly(vinyl alcohol) (PVA), Parteck^®^ MXP 4-88, approximately 88% hydrolyzed, and DZP.

## 2. Materials and Methods

Parteck^®^ MXP, a poly(vinyl alcohol) excipient designed for hot-melt extrusion, was donated by Merck (Darmstadt, Germany), while diazepam was provided by Generis^®^ Farmacêutica S.A. (Lisbon, Portugal). Hydrochloric acid (HCl) (Honeywell FlukaTM, Fisher Scientific, Charlotte, NC, USA) was used to prepare the 0.1 N HCl medium. Commercially available DZP tablets with 5 and 10 mg DZP contents (Diazepam Labesfal, Generis^®^ Farmacêutica, S.A., Lisbon, Portugal) were characterized in terms of mass and dimensions.

### 2.1. Preparation of Placebo and DZP-Loaded Filaments

A DZP-loaded formulation was prepared by mixing 90% (*w*/*w*) PVA and 10% (*w*/*w*) DZP in a mortar for 15 min. The blend was, thereafter, sieved (500 μm) to remove agglomerates. The placebo formulation (100% PVA) and DZP-loaded formulation were extruded (100 g) in a single-screw extruder (Noztek Pro, Noztek, Shoreham, UK) equipped with a 1.58 mm diameter die. PVA was processed at a set temperature (210 °C) and screw speed (13 rpm), which allowed the production of filaments with a diameter of 1.75 ± 0.05 mm. To increase the die swelling and obtain filaments within the same diameter range, a DZP-loaded blend was extruded at 205 °C and a screw speed of 15 rpm.

### 2.2. Preparation of Bi-Compartmental Tablets

The filaments were printed using a helloBEEprusa 3D printer (BEEverycreative, Aveiro, Portugal, Figure S1) equipped with a 0.4 mm nozzle. Tablets, both with a single and double compartment, with a total thickness and diameter of 3 and 6.4 mm, respectively, were designed with the Autodesk Fusion 360 (Autodesk, Mill Valley, CA, USA). Printing settings were defined on the Ultimaker Cura 4.10.0 (Ultimaker, Utrecht, The Netherlands) software, which allowed the creation of a .gcode file that was readable by the printer. Settings were the same for both filaments and were as follows: 100% infill, printing speed of 60 mm·s^−1^, layer thickness of 0.2 mm, nozzle temperature of 210 °C, and platform temperature of 50 °C.

For bi-compartmental tablets, the placebo compartment was first printed, and the DZP compartment was printed on top of it afterward. To achieve the desired drug contents (0, 1, 2, 5, 7.5, and 10 mg), the thickness of both the placebo and DZP-loaded formulation was adapted accordingly (Table 1) based on preliminary studies. It was observed that, by using a layer thickness of 0.2 mm, it was not possible to print bi-compartment tablets that had an odd thickness value (e.g., 1.5 mm), as the second compartment to be printed failed to adhere to the previous one. In such cases, the closest even thickness value (a multiple of 0.2 mm) was selected to achieve the desired DZP content on the tablet.

### 2.3. Characterization of Raw Materials, Filaments and Tablets

#### 2.3.1. Assessment of Dimensions

The dimensions of filaments, 3D printed tablets immediately after manufacture, and commercially available tablets (*n* = 10) were measured by a digital caliper. Filaments out of the 1.75 ± 0.05 mm range were discarded.

#### 2.3.2. Mass of Tablets

The mass of printed, immediately after manufacture, and commercially available tablets (*n* = 10) was determined.

#### 2.3.3. Thermal Analysis

Raw materials, physical mixture, filaments and tablets were analyzed by differential scanning calorimetry (DSC) (DSC Q200, TA Instruments, Leatherhead, UK) equipped with a refrigerated cooling system and purged with dry nitrogen at a flow rate of 50 mL·min^−1^. Samples were precisely weighed (±5 mg) and analyzed in Tzero pans at a heating rate of 10 °C·min^−1^. Furthermore, analysis with modulated temperature was also performed at a heating rate of 5 °C·min^−1^, a period of 60 s and an amplitude of 0.79 °C. Raw data were analyzed using the TA Instruments Universal Analysis 2000 v4.5A software, where the glass transition temperature (*T*_g_) was calculated as the midpoint of inflection of the thermal event observed in the respective thermogram and the melting point (*T*_m_) as the onset temperature of the endothermic event.

#### 2.3.4. Diffractometric Analysis

Diffractograms of raw materials, physical mixture, filaments, and tablets were recorded using an X-ray diffractometer (PANalytical, X’Pert PRO, Almelo, The Netherlands) with a CuKa (*λ* = 1.54 Å) radiation source and a defined voltage (40 kV) and current (30 mA). Samples were analyzed in the angular range of 7 < 2*θ* < 35°, using a step size of 0.017° and a counting time of 19.685 s.

#### 2.3.5. Spectroscopic Analysis

Infrared spectra (*n* = 3) of raw materials, physical mixture, and the surface of filaments and tablets were recorded with an FTIR spectrometer (Nicolet iS5, Thermo Fisher Scientific, Waltham, MA, USA) equipped with an ATR accessory over a range of 4000−550 cm^−1^ (16 scans per spectrum).

#### 2.3.6. Microscopic Analysis

To investigate the surface morphology of the filaments and the adhesion between both compartments of the tablet, scanning electron microscopy (SEM) was performed using a JEOL-JSM-S200LV electron microscope (JEOL, Peabody, MA, USA) equipped with a secondary electron detector. A magnification of 15× was used for the observation of filaments, while a magnification of 35× was required for tablets. Prior to analysis, samples were gold coated in a sputtering chamber (JEOL JFC-1200, JEOL, Peabody, MA, USA).

#### 2.3.7. Drug Content

The drug content of tablets (*n* = 6) was determined in a 0.1 N HCl solution after being stirred in 50 mL volumetric flasks for 24 h containing to ensure complete dissolution. Samples were diluted and analyzed spectrophotometrically at 240 nm (U-1900 UV−VIS Spectrophotometer, Hitachi, Tokyo, Japan). 

#### 2.3.8. Drug Release

The in vitro drug release from tablets (*n* = 6) was determined using a type II dissolution apparatus (AT7, Sotax, Aesch, Switzerland) with a paddle speed of 100 rpm. The experiments were performed in 900 mL 0.1 N HCl solution at 37.0 ± 0.5 °C. Samples were withdrawn at pre-set times (between 0 and 180 min), filtered through a 0.22 μm MCE filter (Merck, Burlington, MA, USA) and analyzed spectrophotometrically at 240 nm (U-1900 UV−VIS Spectrophotometer, Hitachi, Tokyo, Japan). Fresh dissolution medium was added after each sample was collected to keep the volume inside the vessel constant. 

### 2.4. Assessment of the Stability of DZP Filaments and Tablets

Both filaments and tablets with DZP were stored in a climate chamber (VC 2033, Vötsch Industrietechnik, Balingen, Germany) at 40 °C/75% relative humidity (RH), a commonly accepted stress condition. Samples were collected after 1 month of storage and analyzed by DSC, and tablets were also analyzed for drug release. The similarity factor (*f*_2_) was calculated to evaluate the similarity between the drug release profiles before and after storage [23].

## 3. Results and Discussion

The use of the FDM technology for the preparation of medicines requires upstream processing for the production of filaments used as feedstock material for the printer. For this purpose, hot-melt extrusion (HME) is the foremost technique used. Thus, the characterization of the raw materials (Figure S2) and filaments is mandatory to assess the printability of the filaments used in the preparation of bi-compartmental DZP-loaded tablets that should remain stable throughout the time of use by the patient.

### 3.1. Preliminary Printing Studies

In the present work, it was aimed to produce tablets with similar dimensions to those currently commercially available. These DZP tablets (doses 5 and 10 mg) had an average mass varying from 100.7 to 118.2 mg and an average thickness and diameter of 3.0 and 6.4 mm, respectively.

It has previously been shown that tablet mass depended on the polymer incorporated in the filaments used to produce the tablets. Samaro and co-workers designed tablets with 10 mm diameter and 4 mm thickness and obtained average masses of 236.5 ± 5.1, 191.0 ± 6.3 and 212.0 ± 5.0 mg for PVA, ABS, and Klucel^TM^ HPC EF-based tablets, respectively. [24]. Therefore, a placebo PVA filament was used in this preliminary study to determine the mass average of a 3D-printed tablet with a thickness of 3 mm and a diameter of 6.4 mm diameter (Table 2). Using this polymer enabled us to select the drug load of the filament used for the production of DZP tablets. Taking into account the production of a 100 mg tablet delivering a maximum DZP content of 10 mg, it was decided to produce filaments with a 10% drug load that would allow us to produce tablets with a single compartment with 10 mg of DZP.

As shown by Pietrzak and co-workers, it is possible to predict the drug content in a printed tablet based on the mass of a series of tablets with increasing volumes [25]. Hence, by keeping the diameter of tablets from the same drug-loaded filament constant, it is possible to change the drug content by only adjusting the thickness of the drug-loaded compartment. A preliminary test indicated that bi-compartmental tablets with a DZP content below 1 mg were hard to produce due to poor adhesion of the small DZP-containing layer (height 0.2 mm) to the placebo compartment as it consisted of a single deposited layer. 

### 3.2. Characterization of Feedstock Materials for HME and FDM

**Figure 1:**
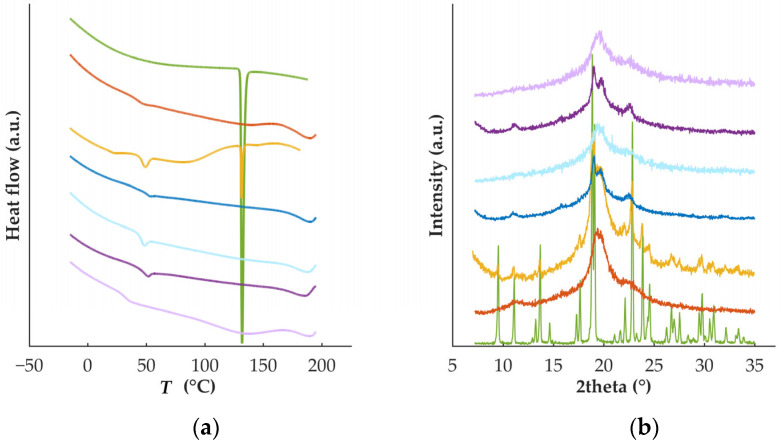
(**a**) Thermograms and (**b**) diffractograms of DZP (green) and PVA (orange) raw materials, physical mixture (yellow) and placebo filaments (dark blue) or tablets (light blue), and DZP-loaded filaments (dark purple) or tablets (light purple).

Thermal analysis on the raw materials (Figure 1a) confirmed the crystalline nature of DZP, with a well-defined melting point (130.7 °C), and the semi-crystalline nature of PVA (*T*_g_ = 42.5 °C and *T*_m_ = 168.5 °C), in good agreement with previous work [26,27]. Data from diffractometry (Figure 1b) was in line with the data collected by calorimetry (DSC). DZP diffractograms presented sharp characteristic peaks at 9.50, 13.68, 18.86, 19.07, 22.85, and 23.87° 2θ, while PVA had broad peaks at 11.23, 19.35 and 22.61° 2θ. Analysis performed on the physical mixture, containing 90% PVA and 10% DZP, revealed the presence of the endothermic melting peak of the drug (at 130.7 °C), suggesting that the drug is not dissolved in the polymer during the heating run. Similarly, the characteristic crystalline peaks of DZP were detected on the physical mixture when analyzed by diffractometry.

**Figure 2:**
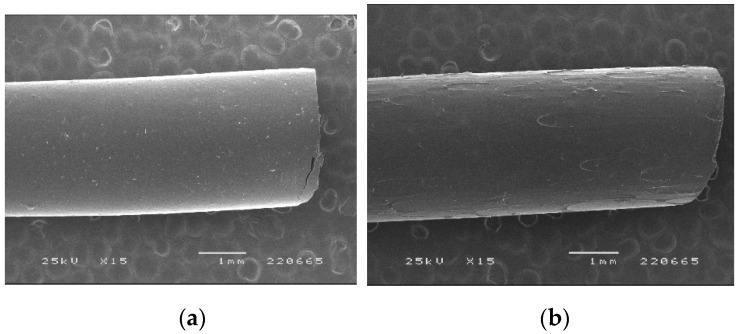
SEM images of PVA-based (**a**) placebo and (**b**) diazepam-loaded (10%) filaments.

When processed by HME under the same conditions as the placebo filament, diazepam showed a plasticizing effect on PVA, which reduced the extrudate’s diameter due to the lower viscosity of the processed material [28]. Since the diameter of the filaments was too small to enable the proper feeding of the printer, the speed of the screw speed was increased, and the processing temperature decreased. Visually, filaments were transparent and presented a smooth surface. SEM analysis (Figure 2) was performed to evaluate whether the incorporation of DZP in the formulation had an influence on the morphology of the filament surface.

The incorporation of DZP in the formulations negatively affected the smoothness of filament surfaces with small defects detected by SEM. Verstraete and co-workers have shown that the incorporation of a drug in polyurethanes-based formulations had a negative impact on the morphology of the filaments produced [29]. Fortunately, feeding the printer with such filaments was possible without major issues, and proper flow toward the printer nozzle was observed. 

Thermograms of the filaments showed the complete conversion of crystalline DZP to its amorphous counterpart based on the absence of a melting event (Figure 1a, dark purple line). Concomitantly, diffractograms confirmed the formation of an amorphous solid dispersion (ASD) during HME, as no peaks characteristic of the crystalline drug were detected. The ASD presented a single *T*_g_ (*T*_g_ = 49.1 °C), suggesting that a homogeneous system was obtained and that DZP had a slight plasticizing effect on PVA-based filament (*T*_g_ = 51.2 °C) and supporting the plasticizing effect of the drug on the polymer observed during processing by HME.

**Figure 3:**
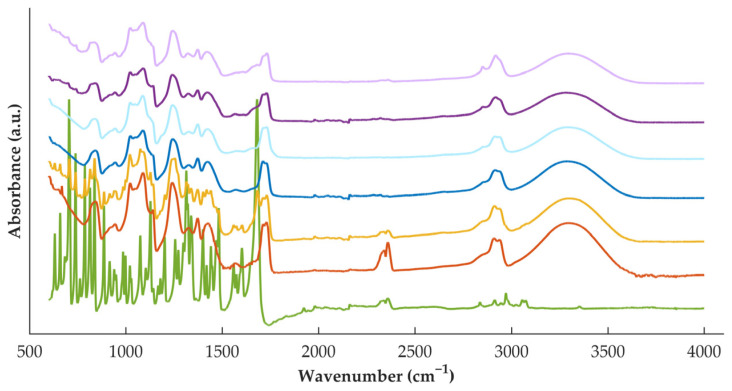
FTIR spectra of DZP (green) and PVA (orange) raw materials, physical mixture (yellow) and placebo filaments (dark blue) or tablets (light blue), and DZP-loaded filaments (dark purple) or tablets (light purple).

During processing, high shear and temperatures are applied to the materials, and, therefore, the interactions between the polymer and the drug were investigated by FTIR analysis (Figure 3). For DZP raw material, the characteristic band at 1681 cm^−1^ can be attributed to the imine groups (C=N), while the one at 1600 cm^−1^ to the conjugated alkene (C=C). This spectrum is similar to the one observed in previous work [30,31]. PVA spectra present a large band in the range of 3550–3000 cm^−1^ attributed to the O–H from the intermolecular and intramolecular hydrogen bonds and a band at 1141 cm^−1^ due to the C–O groups. When analyzing the spectra from the physical mixture, it is possible to detect the characteristic bands of both materials, suggesting that no interactions were established by the simple mixing.

When considering the placebo filament, it is possible to observe that its spectra resemble well the spectra of the polymer as raw material (powder). On the other hand, DZP-loaded filament does not resemble the spectra of the physical mixture, with some bands characteristic of the API disappearing on the filament spectra. This observation suggested the establishment of interactions when the materials were processed under stress conditions, which may have contributed to the formation of the ASD detected by calorimetry and diffractometry.

### 3.3. Characterization of Printed Tablets

**Figure 4:**
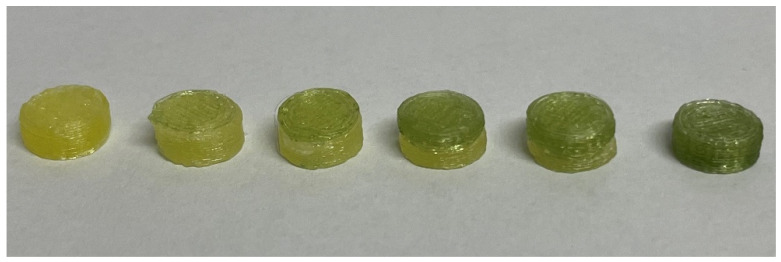
Image of tablets with increased diazepam (DZP) contents: 0, 1, 2, 5, 7.5, and 10 mg (from left to right). The tablet on the left was prepared with a placebo filament only, whereas the tablet on the right side was prepared using the DZP-loaded filament only. In the bi-compartmental tablets, the lower compartment was made of a placebo filament, whereas the DZP-loaded filament was applied on the top.

When preparing bi-compartmental tablets, proper adhesion and alignment between both compartments are major challenges. It was observed during the first printing trials that the adhesion of the layers for the second compartment depended on the proper alignment of both nozzles. At the optimized alignment, the printing of bi-compartmental tablets was possible (Figure 4). 

When considering the administration of such tablets to patients, it is suggested to add a dye during the extrusion of both formulations to ensure the same color is obtained for the placebo and drug-loaded compartments. In the present work, to better differentiate the different tablets, no dye was added.

**Figure 5:**
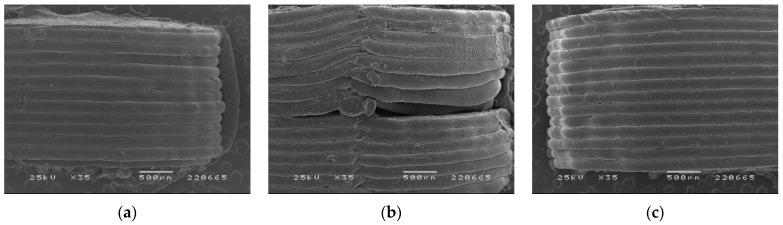
SEM images of printed tablets with (**a**) 0, (**b**) 5 and (**c**) 10 mg of diazepam.

To visualize the morphology of the tablets and the adhesion between layers within the tablets, SEM images were collected from tablets containing different DZP loads (Figure 5). Tablets obtained by printing a single filament (Figure 5a,c) presented an excellent layer definition, with no gaps between the different layers, whereas bi-compartmental tablets showed a small gap at the corner of the interface between the two compartments (Figure 5b, right). This defect only occurred when the first layer of the second compartment was printed and is likely related to some inertia of the first material coming out of the nozzle, particularly because the left side of the same layer in the tablet merged properly with the previous layer (Figure 5b, left).

Paramount for the project is the required similarity of morphology, masses, and sizes of tablets to promote patient compliance with the therapy by avoiding the perception of differences between tablets regarding the DZP dose delivered. For the six doses prepared, the average mass of tablets varied from 104.1 to 120.9 mg, which is similar to the mass range observed for the commercially available tablets (100.7–118.2 mg). Furthermore, each dose complied with the European Pharmacopeia requirements for tablet mass uniformity [32]. Regarding the dimensions of the tablets (Table 2), tablets made of a single filament (0 and 10 mg DZP dose) presented a similar thickness to the one designed on the CAD model, while bi-compartmental tablets were thicker than the CAD settings. This contradicts the findings of previous works, which have shown a similar or lower thickness than the ones designed on the CAD software [11,12]. The difference is likely related to the gap observed in SEM images of bi-compartmental tablets that led to an increased overall thickness of these tablets. Figure S3 summarizes the dispersion of data related to the mass (a), thickness (b), diameter (c) and DZP content (d) of 3D-printed tablets.

The average drug content of tablets varied between 99.3 and 105.5% of the theoretical content (Table 3), indicating that FDM 3D printing was able to accurately control the drug content during the production of tablets and highlighting the potential of this technology for the production of tablets with a continuous drug dose. 

**Figure 6:**
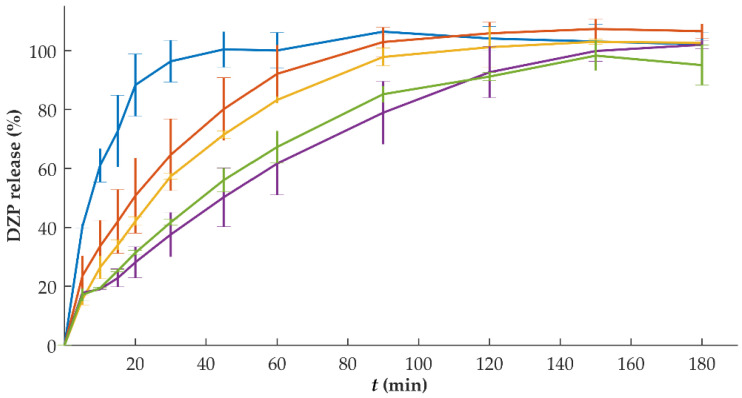
In vitro release (mean ± sd, *n* = 6) of diazepam (DZP) from printed tablets with a dose of 1 (blue), 2 (orange), 5 (yellow), 7.5 (purple) and 10 (green) mg.

The release rate of DZP was faster than tablets with a lower drug content (Figure 6). This might be attributed to the thicker DZP compartments, which took longer to dissolve for the tablets with higher DZP content. Tablets with 1 mg of DZP were able to release the drug within 30 min, while tablets with 10 mg of DZP required 150 min to release the entire dose. For the lowest dose, DZP was mostly at the surface of the tablet (top side of the tablet), whereas the DZP in higher doses was present in layers not immediately exposed to the dissolution medium.

Based on this observation, several modifications (e.g., different geometry for the same total volume of tablet) could be applied to promote similar percentages of drug release per unit of time. For instance, a higher SA/V ratio of the drug-loaded compartment increases the drug release rate. This would have been relevant for intermediate doses (5 and 7.5 mg) in which the drug-loaded compartment could have been divided into two to generate a tri-compartmental tablet, with the placebo compartments in the middle, and the DZP-loaded compartments covering it at the bottom and top, increasing the surface area in contact with the dissolution medium. Another possibility was tested by Buyukgoz et al. by printing the drug-loaded filaments peripherally with the placebo portion as the core of the tablet [16]. On the other hand, modifications to the formulation could also be tested, like the addition of disintegrants to the formulation. Such modification would approach the release of the drug from these tablets with the release of commercially available ones, in which DZP was completely released within 5 min (results not shown).

To investigate the solid state of the drug on the printed tablets, DSC and XRD experiments were performed (Figure 1). Similarly to what was observed for the filaments, DZP was in the amorphous form. The second heat treatment did not destabilize the ASD formed during HME. However, a decrease in the *T*_g_ in tablets (*T*_g_ = 31.2 °C) was observed in comparison with the *T*_g_ in filaments (*T*_g_ = 49.2 °C). Such a decrease in the *T*_g_ may have a negative impact on the stability of the ASD upon storage.

When comparing the FTIR spectra (Figure 3) of filaments and tablets (darker and light curves, respectively) for both placebo and DZP-loaded formulations, no changes were detected, with the spectra resembling each other. Such observation indicates that no new interactions were established when applying a second heat treatment to the formulation, and no previously established interactions were broken.

### 3.4. Stability of Filaments and Tablets

**Figure 7:**
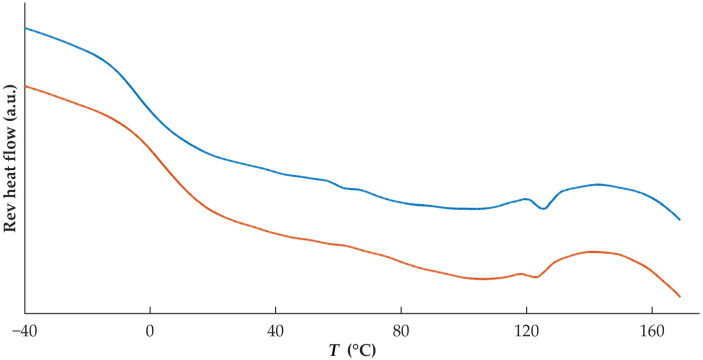
Thermograms of DZP-loaded filament (blue) and tablet (orange) after 1 month of storage at 40 °C/75% RH.

Both filaments and tablets containing DZP were stored at 40 °C/75% RH for 1 month, and thermograms (Figure 7) from collected samples (filaments and tablets) enabled the identification of a small melting event with onset at 121.3 and 119.0 °C for filament and tablet samples, respectively. This could be due to the recrystallization of DZP, even occurring at a lower temperature than the *T*_m_ of pure DZP. Melting point depression of drugs has been attributed to interactions at the molecular level between two materials in the formulation [33,34]. A reduction of the *T*_g_ to −4.4 and 3.2 °C for filaments and tablets, respectively, was also detected. As all samples were exposed to high levels of humidity, water may have acted as a plasticizer. 

**Figure 8:**
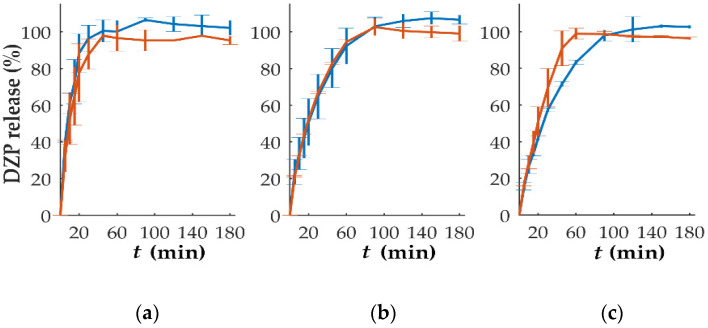

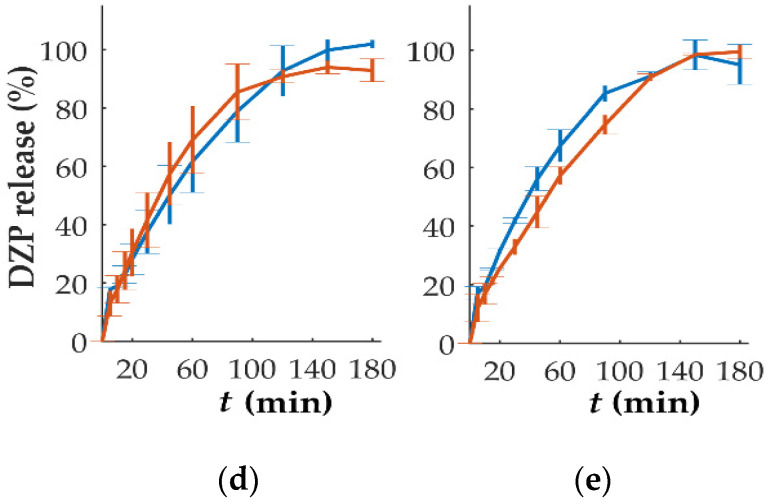
In vitro release (mean ± sd, *n* = 6) of diazepam (DZP) from printed tablets immediately after production (blue) and after 1 month of storage at 40 °C/75% RH (orange), with a dose of (**a**) 1, (**b**) 2, (**c**) 5, (**d**) 7.5 and (**e**) 10 mg.

In vitro dissolution studies performed on tablets with different DZP contents stored at high temperatures and humidity have been performed (Figure 8). The comparison based on the *f*_2_ analysis showed that the release from tablets with 10 mg could be considered similar (*f*_2_ = 54.8), even with a slightly slower drug release, when compared to the release observed immediately after production, was observed. This could be attributed to drug recrystallization, detected by DSC (Figure 6), that might have delayed the release. For tablets with 1, 2 and 7.5 mg of DZP, no significant differences (*f*_2_ = 60.7, 81.9 and 65.5 for 1, 2 and 7.5 mg, respectively) on the release profile were observed when compared to the profile obtained for the same tablets immediately after production. On the other hand, tablets with 5 mg DZP showed a faster drug release after storage under stress conditions, with the similarity factor *f*_2_ of 47.3. Although no explanation could be found for this phenomenon, it is possible that the two compartments composing the bi-compartmental tablet had some degree of detachment during dissolution that led to an increase in the SA/V ratio of the DZP-loaded compartment, accelerating the release of the drug.

No drug degradation seemed to have occurred during the storage, as the release between 97.7 and 102.5% of the expected drug content was achieved during the dissolution testing.

## 4. Conclusions

To avoid withdrawal problems from diazepam therapy, a slow tapering of the dose should be done. The present work demonstrated the usefulness of FDM 3D printing to effectively produce tablets with continuous diazepam doses that could be used for withdrawal from DZP. Dual-nozzle FDM was able to produce tablets with similar masses and dimensions while modifying the drug content, making the changes imperceptible for the patient. Tablets with diazepam contents as low as 1 mg could be produced, avoiding patients needing to split tablets to achieve lower drug contents.

Stability tests on the tablets stored under stress conditions for one month have shown the inability of PVA to prevent the recrystallization of diazepam, which influenced, although not significantly, the drug release rate at the highest DZP content (made of a single compartment).

## Figures and Tables

**Table 1:** Thickness of the diazepam (DZP) compartment to achieve the desired DZP content.

DZP Target Content (mg)	Thickness DZP-Compartment (mm)	
	Based on Calculation	Designed in CAD
1	0.3	0.4
2	0.6	0.6
5	1.5	1.6
7.5	2.25	2.2
10	3.0	3.0

**Table 2:** Mass, thickness and diameter (*n* = 10) of placebo and diazepam (DZP)-loaded tablets.

DZP Target Content (mg)	Mass (mg)	Thickness (mm)	Diameter (mm)
0.0	104.1 ± 5.4	2.9 ± 0.1	6.5 ± 0.2
1.0	117.2 ± 4.0	3.2 ± 0.1	6.5 ± 0.3
2.0	120.9 ± 3.4	3.2 ± 0.1	6.6 ± 0.2
5.0	110.8 ± 5.6	3.3 ± 0.1	6.3 ± 0.1
7.5	112.5 ± 4.2	3.3 ± 0.0	6.3 ± 0.0
10.0	104.4 ± 5.6	3.0 ± 0.2	6.4 ± 0.2

**Table 3:** Diazepam (DZP) content in tablets (*n* = 6).

DZP Content (mg)	
Target	Observed
1.0	1.05 ± 0.04
2.0	2.10 ± 0.06
5.0	5.12 ± 0.38
7.5	7.45 ± 0.21
10.0	10.14 ± 0.38

## Data Availability

Not applicable.

## Supplementary Materials

The following supporting information can be downloaded at: https://www.mdpi.com/article/10.3390/pharmaceutics15020538/s1, Figure S1: Schematic representation of the dual-nozzle printer system used in the current study (helloBEEprusa 3D printer, BEEverycreative, Aveiro, Portugal); Figure S2: Chemical structure of (a) diazepam and (b) poly(vinyl alcohol); Figure S3: Data dispersion scheme of (a) mass, (b) thickness, (c) diameter and (d) DZP content of 3D printed tablets.
