# Supplementary material for: Production of Bi-Compartmental Tablets by FDM 3D Printing for the Withdrawal of Diazepam
Source: Pharmaceutics. 2023 Feb 6;15(2):538. doi: 10.3390/pharmaceutics15020538 (PMC9960133; doi:10.3390/pharmaceutics15020538)
Supplement: Supplementary file 1 [file pharmaceutics-15-00538-s001.zip › pharmaceutics-2160701-supplementary.pdf]

pharmaceutics

MDPI

Supplementary materials: Production of Bi-Compartmental Tablets by FDM 3D Printing for the Withdrawal of Diazepam

Joana Macedo, Rita Marques, Chris Vervaet and João F. Pinto

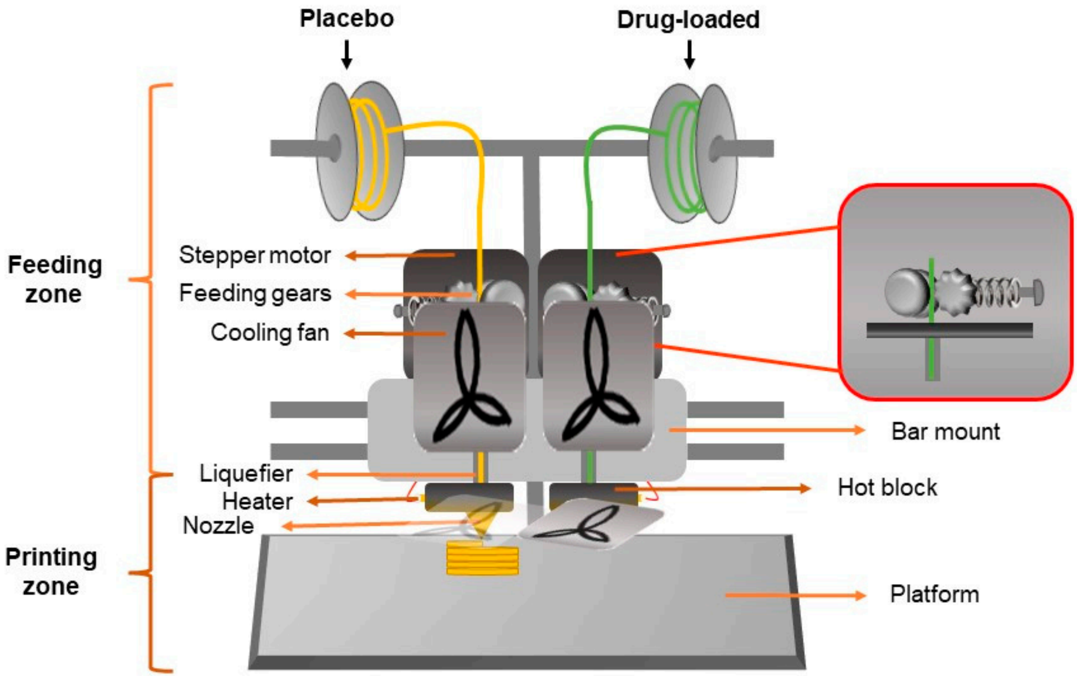

Figure S1. Schematic representation of the dual-nozzle printer system used in the current study (helloBEEprusa 3D printer, BEEverycreative, Aveiro, Portugal).

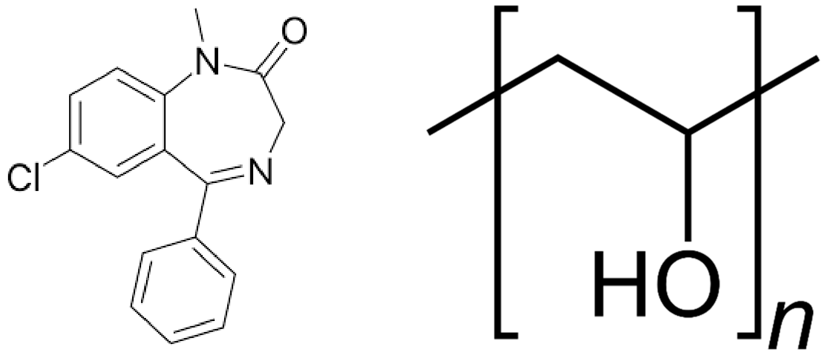

(**a**) (**b**)

Figure S2. Chemical structure of (**a**) diazepam and (**b**) poly(vinyl alcohol).

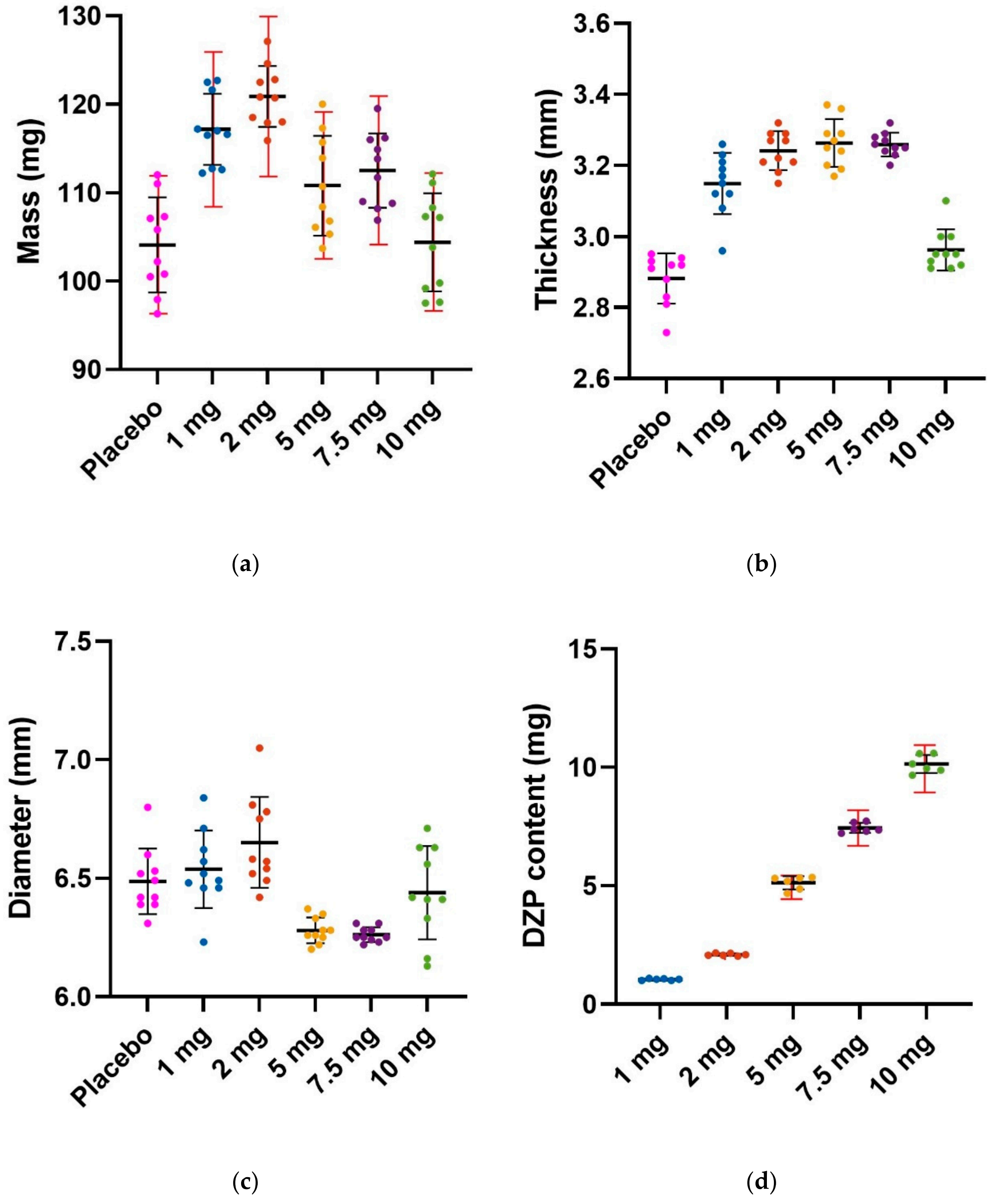

Figure S3. Data dispersion scheme of (**a**) mass, (**b**) thickness, (**c**) diameter and (**d**) DZP content of 3D printed tablets.

The mean and standard deviation are represented in black. For (**a**) and (**d**), the controls, according to the European Pharmacopoeia, are also presented in red. For the mass, this is 7.5% of the mean value and for the drug content, the control is 10% of the theoretical content.
